# The radiologist’s role in detecting systemic anticancer therapy-related interstitial lung disease: an educational review

**DOI:** 10.1186/s13244-024-01771-z

**Published:** 2024-08-01

**Authors:** Julien Dinkel, Nikolaus Kneidinger, Paolo Tarantino

**Affiliations:** 1 Department of Radiology, University Hospital LMU Munich, Munich, Germany; 2 Comprehensive Pneumology Center (CPC-M), Member of the German Center for Lung Research (DZL), Munich, Germany; 3 Department of Medicine V, Comprehensive Pneumology Center (CPC-M), Member of the German Center for Lung Research (DZL), Munich, Germany; 4 Division of Pulmonology, Department of Internal Medicine, Medical University of Graz, Graz, Austria; 5 Breast Oncology Center, Dana-Farber Cancer Institute, Boston, MA USA; 6 Harvard Medical School, Boston, MA USA; 7 Department of Oncology and Onco-Hematology, University of Milan, Milan, Italy

**Keywords:** Lung diseases, Interstitial, Drug-related side effects and adverse reactions, Tomography (x-ray computed), Neoplasms

## Abstract

**Abstract:**

Systemic anticancer therapies (SACTs) are the leading cause of drug-induced interstitial lung disease (ILD). As more novel SACTs become approved, the incidence of this potentially life-threatening adverse event (AE) may increase. Early detection of SACT-related ILD allows for prompt implementation of drug-specific management recommendations, improving the likelihood of AE resolution and, in some instances, widening the patient’s eligibility for future cancer treatment options. ILD requires a diagnosis of exclusion through collaboration with the patient’s multidisciplinary team to rule out other possible etiologies of new or worsening respiratory signs and symptoms. At Grade 1, ILD is asymptomatic, and thus the radiologist is key to detecting the AE prior to the disease severity worsening. Planned computed tomography scans should be reviewed for the presence of ILD in addition to being assessed for tumor response to treatment, and when ILD is suspected, a high-resolution computed tomography (HRCT) scan should be requested immediately. An HRCT scan, with < 2-mm slice thickness, is the most appropriate method for detecting ILD. Multiple patterns of ILD exist, which can impact patient prognosis. The four main patterns include acute interstitial pneumonia / acute respiratory distress syndrome, organizing pneumonia, hypersensitivity pneumonitis, and non-specific interstitial pneumonia; their distinct radiological features, along with rarer patterns, are discussed here. Furthermore, HRCT is essential for following the course of ILD and might help to determine the intensity of AE management and the appropriateness of re-challenging with SACT, where indicated by drug-specific prescribing information. ILD events should be monitored closely until complete resolution.

**Critical relevance statement:**

The incidence of potentially treatment-limiting and life-threatening systemic anticancer therapy-related interstitial lung disease (SACT-related ILD) events is likely increasing as more novel regimens become approved. This review provides best-practice recommendations for the early detection of SACT-related ILD by radiologists.

**Key Points:**

- Radiologists are crucial in detecting asymptomatic (Grade 1) ILD before severity/prognosis worsens.
- High-resolution computed tomography is the most appropriate method for detecting ILD.
- Drug-induced ILD is a diagnosis of exclusion, involving a multidisciplinary team.
- Familiarity with common HRCT patterns, described here, is key for prompt detection.
- Physicians should highlight systemic anticancer therapies (SACTs) with a known risk for interstitial lung diseases (ILD) on scan requisitions.

**Graphical Abstract:**

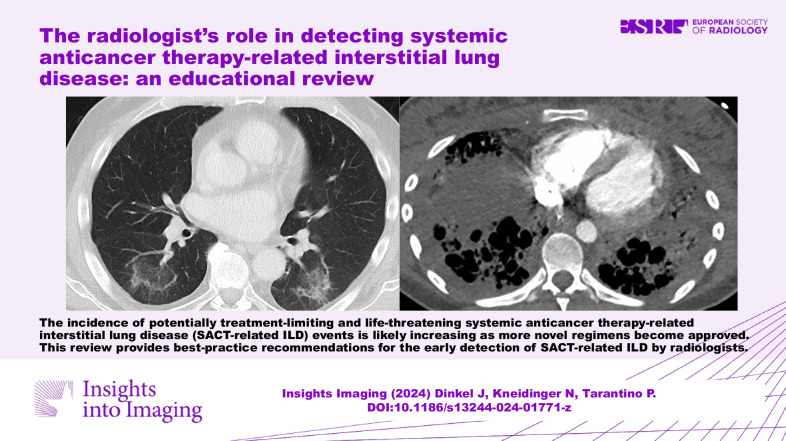

## Background

### Introduction

Interstitial lung disease (ILD) is a heterogeneous group of lung disorders, including pneumonitis, that manifest as inflammation and/or fibrosis of the lungs [1, 2]. ILD can have a broad range of etiologies, occurring because of hypersensitivity; exposure to specific toxic metals; or autoimmune, inflammatory, and genetic disorders; or secondary to smoking-related disorders [3]. In Europe, most cases of ILD are idiopathic or related to sarcoidosis, with between 11.5% and 38.6% of cases having no identifiable cause [4]. A subset of ILD events result from exposure to drugs [3, 5]; systemic anticancer therapies (SACTs) are the leading cause of drug-induced ILD [6]. As the number of novel anticancer agents and indications for existing anticancer agents associated with ILD increases, SACT-related ILD is expected to become more prevalent across cancer types [7]. A recently published position paper from the Fleischner Society identified and answered specific questions regarding the diagnostic criteria for and management of drug-induced ILD in patients with cancer receiving molecular targeting agents and immune checkpoint inhibitors (ICIs) [8]. In this review, the role of the radiologist in detecting SACT-related ILD is outlined, and the authors provide best-practice recommendations on the most appropriate imaging criteria and key radiological abnormalities to be vigilant for when monitoring patients.

### The importance of early detection of SACT-related ILD

The severity of ILD events is typically graded from 1 to 5 using the Common Terminology Criteria for Adverse Events (CTCAE) version 5.0 [7, 9]. Grade 1 ILD is asymptomatic, and Grade ≥ 2 events are symptomatic (Table 1) [9]. Fine crackles on chest auscultation and indications of interstitial changes on chest imaging—as well as fever or new or worsening respiratory symptoms, including dyspnea, cough, and hypoxia at rest or with exertion—indicate that a patient may have ILD [10, 11].

**Table 1:** CTCAE version 5.0 grading of ILD, defined as focal or diffuse inflammation affecting the lung parenchyma [9]

	CTCAE grade				
	1	2	3	4	5
**CTCAE definition**	Asymptomatic with clinical or diagnostic (e.g., radiological) findings only; intervention not indicated	Symptomatic; instrumental ADLs are limited, and medical intervention indicated	Severe symptoms; self-care ADLs are limited, and oxygen is indicated	Life-threatening respiratory compromise; urgent intervention is needed (e.g., tracheotomy or intubation)	Death related to AE

*ADL* activity of daily living, *AE* adverse event, *CTCAE* Common Terminology Criteria for Adverse Events, *ILD* interstitial lung disease

Time to diagnosis of SACT-related ILD has clinical and therapeutic consequences. Early diagnosis allows for prompt treatment of ILD with corticosteroids as recommended in the prescribing information (PI) of some SACTs [12, 13], increasing the likelihood of complete resolution of the adverse event (AE) [7], and also providing the opportunity to rechallenge patients who are eligible for resuming their treatment upon resolution of the AE, per drug-specific PI [12, 13]. Urgent reporting of ILD to a patient’s treating physician can also prevent redosing until AE resolution, reducing the risk of escalating severity.

As Grade 1 ILD is defined as an asymptomatic event, with radiological findings only [9], the radiologist is key for the early identification of ILD via proactive monitoring for manifestations before symptom onset. Proactive communication between treating physicians and radiologists provides more opportunity for prompt detection of SACT-related ILD. Treating physicians should indicate on scan requisitions when a patient is receiving SACTs with a relevant risk for ILD. Planned computed tomography (CT) scans should be assessed for the presence of ILD in addition to treatment response [14], especially when tumor assessments and redosing are scheduled for the same day. Where ILD is suspected, radiologists should be consulted, and a joint review of scans can be considered when the patient is being treated with a drug known to increase the risk of developing ILD. During routine CT scans for tumor response, radiologists should inform the ordering physician when ILD is suspected. When diagnosing SACT-related ILD, a multidisciplinary approach should be used to rule out other causes [15]. A pulmonary consultation assessing the potential impact of suspected lung damage with a pulmonary function test is also recommended for suspected ILD, particularly in symptomatic cases [16].

## Pathogenesis of SACT-related ILD

Two basic mechanisms, direct cytotoxic and immune-mediated lung injury, are commonly accepted as the likely underlying processes for SACT-related ILD, despite the exact pathogenesis not being completely understood [17, 18]. Direct cytotoxic effects may manifest via multiple pathways resulting in damage to alveolar Type I epithelial cells, airway epithelial cells, or vascular endothelial cells [17, 19]. Neutrophilia has been observed in patients with SACT-related ILD [20], and has been theorized to trigger the release of neutrophil extracellular traps, inducing pulmonary fibrosis [21]. Metabolism of SACTs in the lungs may result in the release of highly cytotoxic reactive oxygen species leading to pulmonary injuries [19]. Several theories have been posed to explain the association between antibody-drug conjugates (ADCs) and ILD, including the target-dependent uptake and catabolism of ADCs or cytotoxic effects resulting from potential uptake and catabolism in off-target lung epithelial cells [22, 23]. The bystander effect, related to the membrane-permeable tetrapeptide-based cleavable linker present in some ADCs, may also be a mechanism for cytotoxic injury [23]. As ILD has been observed in patients receiving ADCs with a range of different therapeutic targets, including human epidermal growth factor receptor 2 (HER2), folate receptor α, and trophoblast cell surface antigen 2, it is likely that the lung toxicity associated with ADCs is not related to any specific target protein [24]. Some SACTs may cause amplified autoimmune responses and subsequent inflammation and immune-mediated lung injury [19]. Immune cells may be activated by SACTs that mimic antigens through non-recognition of the drug triggering pro-inflammatory signals, or via the hapten hypothesis [17, 19], where drugs are proposed to modify cellular proteins and generate novel molecules that stimulate multiple immune pathways [25].

## Risk factors for and incidence of SACT-related ILD

The risk of developing ILD has been associated with various types of SACT, with warnings in the labels of specific ADCs, anti-programmed cell death protein 1 (PD-1) drugs, cyclin-dependent kinase 4/6 inhibitors (CDK4/6i), mammalian target of rapamycin (mTOR) inhibitors, and tyrosine kinase inhibitors (TKIs) [12, 13, 26–30]. For example, a meta-analysis of 12876 patients with solid tumors across 23 randomized controlled trials (RCTs) found that those receiving the PD-1 inhibitors nivolumab or pembrolizumab were associated with a significantly increased risk of developing any-grade and Grade ≥ 3 ILD compared with those receiving chemotherapy (risk ratio 5.17 [95% confidence interval (CI) 2.82, 9.47], *p* < 0.001, and 4.14 [95% CI 1.82, 9.42], *p* < 0.001, respectively) [31]. Similarly, another meta-analysis investigated the incidence of ILD among patients with various types of cancer receiving the CDK4/6i abemaciclib, palbociclib, or ribociclib across 12 Phase 2/3 clinical trials (*N* = 16060) [32]. The study found that in patients receiving CDK4/6i, the risks of developing any-grade ILD were doubled and the risk of developing Grade ≥ 3 ILD was tripled compared with controls (Peto odds ratio 2.12 [95% CI 1.57, 2.86], *p* < 0.00001, and 3.22 [95% CI 1.28, 8.09], *p* = 0.01, respectively) [32]. A meta-analysis of results from 12 RCTs investigated the association between patients with breast or gastric cancer treated with HER2-directed ADCs, including trastuzumab emtansine (T-DM1), trastuzumab deruxtecan (T-DXd), and trastuzumab duocarmazine, and the risk of developing ILD compared with controls [33–45]. Among patients treated with HER2-directed ADCs, there was more than double the risk of developing both any-grade and Grade ≥ 3 ILD compared with controls receiving the standard-of-care therapies lapatinib plus chemotherapy, or trastuzumab alone or in combination with pertuzumab and/or chemotherapy (Peto odds ratio 2.62 [95% CI 1.71, 4.04], *p* < 0.0001, and 2.82 [95% CI 1.07, 7.42], *p* = 0.04, respectively) [33].

Although the risk of developing SACT-related ILD may vary between therapeutic agents, patients with male sex at birth, an Eastern Cooperative Oncology Group performance status (ECOG PS) > 1, and/or those with advanced age have been found to be at greater risk of developing SACT-related ILD when treated with various SACTs than their counterparts [6, 46–48]. Pre-existence of interstitial lung abnormalities (ILAs), defined as both fibrotic and non-fibrotic features on CT scans, and pre-existence of lung diseases may also be risk factors for patients receiving SACTs [47–51]. A retrospective, blinded, single-center cohort study found that in patients with non-small cell lung cancer receiving ICIs (*N* = 475), the risk of developing Grade ≥ 2 SACT-related ILD was doubled among those with pre-existing ILAs compared with their peers without ILAs (odds ratio 2.2 [95% CI 1.03, 4.50], *p* = 0.041) [51]. Several retrospective analyses investigating patients receiving TKIs or chemotherapeutic agents have demonstrated that those with pre-existing lung diseases, including concurrent or pre-existing chronic obstructive pulmonary disease, emphysema, and ILD, were at an increased risk of developing SACT-related ILD compared with their peers [47–50]. The overall incidence of SACT-related ILD in clinical trials ranges from 0.2% to 15.4%, depending on the treatment (Table 2).

**Table 2:** Incidence and mortality rates of ILD in patients treated with various SACTs in clinical trials

Target	Drug^a^	Number of studies (number of patients receiving study drug)	Study phase(s)	Cancer types	Any-grade ILD event, n (%)	Any Grade ≥ 3 ILD event, n (%)	Any Grade 5 ILD event, n (%)
**Anti-HER2**	T-DM1 [101]	7^b^ (884)	2/3	Breast	10 (1.1)	3 (0.3)	1 (0.1)
	T-DXd [94]	9 (1150)	1/2	Breast, colorectal, gastric, lung, other	177 (15.4)	40 (3.5)	25 (2.2)
**Anti-HER2/EGFR**	Lapatinib^c^ [102–104]	3 (4413)	2/3/EAP	Breast	7 (0.2)	NR	0
**Anti-mTOR**	Everolimus [105, 106]	2 (409)	3	Gastrointestinal, lung, pancreatic	67 (6.1)	9 (2.2)	1 (0.2)
**Anti-CDK4/6**	Abemaciclib^d^ [107, 108]	2 (2946)	2/3	Breast	82 (2.8)	13 (< 0.1)	2 (< 0.1)
	Palbociclib^e^ [109, 110]	4 (3352)	2/3	Breast	28 (0.8)	NR	0
	Ribociclib^f^ [111, 112]	2 (838)	3	Breast	7 (0.9)	1 (0.1)^g^	NR
**Anti-PD-1**	Nivolumab^h^ [113–115]	3 (1465)	1–3	Melanoma, SCLC	63 (4.3)	13 (0.9)	1 (0.1)
	Pembrolizumab [116–119]	5 (2014)	2/3	Melanoma, NSCLC, urothelial	63 (3.1)	27 (1.3)	6 (0.4)^i^
**Anti-PD-L1**	Atezolizumab^j^ [120]	3 (1577)	3	NSCLC	88 (5.7)	25 (1.6)	NR

^a^ Patient received systemic anticancer monotherapies unless stated otherwise; ^b^ Results are reported from a pooled analysis of six clinical trials and one extension study [101]; ^c^ Patients in two of the studies received lapatinib plus chemotherapy, with patients in the Phase 2 study receiving lapatinib monotherapy [102–104]; ^d^ Patients across the two studies received abemaciclib plus ET, with or without trastuzumab [107, 108]; ^e^ All four studies investigated palbociclib plus ET [109, 110]; ^f^ Patients received ribociclib plus an ET in both studies [111, 112]; ^g^ Studies only reported the incidence of any-grade ILD events and events that were Grades 3/4; Grade 5 events were not reported [111, 112]; ^h^ Across the three studies patients either received nivolumab monotherapy or nivolumab in combination with ipilimumab [113–115]; ^i^ Patients from one study excluded from calculation as this did not report the number of Grade 5 events [119]; ^j^Patients in the treatment groups received either atezolizumab plus carboplatin/cisplatin plus (nab-)paclitaxel/pemetrexed or atezolizumab plus bevacizumab plus carboplatin and paclitaxel [120]

*CDK4/6* cyclin-dependent kinase 4/6, *EAP* early access program, *EGFR* epidermal growth factor receptor, *ET* endocrine therapy, *HER2* human epidermal growth factor receptor 2, *ILD* interstitial lung disease, *mTOR* mammalian target of rapamycin, *NR* not reported, *NSCLC* non-small cell lung cancer, *PD-1* programmed cell death protein 1, *PD-L1* programmed cell death ligand 1, *SACT* systemic anticancer therapy, *SCLC* small cell lung cancer, *T-DM1* trastuzumab emtansine, *T-DXd* trastuzumab deruxtecan

## Detection of SACT-related ILD

### Recommendations for monitoring patients receiving SACTs associated with a risk of developing ILD

The role of a baseline CT examination should go beyond oncological staging, enabling assessment of lung parenchyma before SACTs are introduced. In addition to treatment evaluation during follow-up CT scans, those receiving SACTs known to be associated with ILD should be screened for any potential signs of ILD. The incidental appearance of pulmonary pathology on CT scans should be investigated by a radiologist.

When ILD is suspected, a high-resolution computed tomography (HRCT) scan should be ordered [11, 15]. HRCT is more sensitive than standard CT scans and enables the extent of lung involvement to be assessed [7, 19]. Thin slices should be used (< 2-mm slice thickness), along with high-resolution reconstruction kernel, improving spatial resolution and allowing subtle reticular and nodular changes to be distinguished that would not be observable with a conventional chest CT scan [52, 53]. HRCT scans are routinely obtained at full inspiration; however, expiratory images may be helpful for determining the cause of a mosaic attenuation pattern [53]. The highest pitch and shortest rotation time feasible should be used to reduce the time for image acquisition and minimize the likelihood of movement introducing artifacts [54], especially as patients are often dyspneic and may not be able to hold their breath for long periods of time [55].

Once SACT-related ILD has been diagnosed based on radiological findings in combination with the results of other multidisciplinary team (MDT) testing, close monitoring using HRCT scans should continue until resolution of ILD, regardless of severity; this includes after drug discontinuation [14, 15]. Frequency of repeat HRCT scans should be individualized to each patient, depending on a multitude of clinical factors, including but not limited to the causative agent, pattern of ILD, grade of the event, and baseline risk factors. In general, close follow up is recommended for all cases of ILD, with repeat scans and clinical assessments within 1–2 weeks from diagnosis for low-grade cases (Grade ≤ 2), and within a few days of diagnosis for high-grade cases (Grade ≥ 3).

### Presentation patterns of SACT-related ILD

The prognosis of SACT-related ILD varies not only by grade of the AE and the associated drug a patient receives, but may also depend on the radiological pattern [7]. Though there are no specific radiological features specific to ILD [19], patterns of presentation have been defined by the joint American Thoracic Society (ATS) and European Respiratory Society (ERS) guidelines [56]. The four main patterns that commonly present for SACT-related ILD include organizing pneumonia (OP), hypersensitivity pneumonitis (HP), acute interstitial pneumonia / acute respiratory distress syndrome (AIP/ARDS), and non-specific interstitial pneumonia (NSIP) [57].

#### Patterns with areas of high attenuation on HRCT

##### OP pattern

**Fig. 1:**
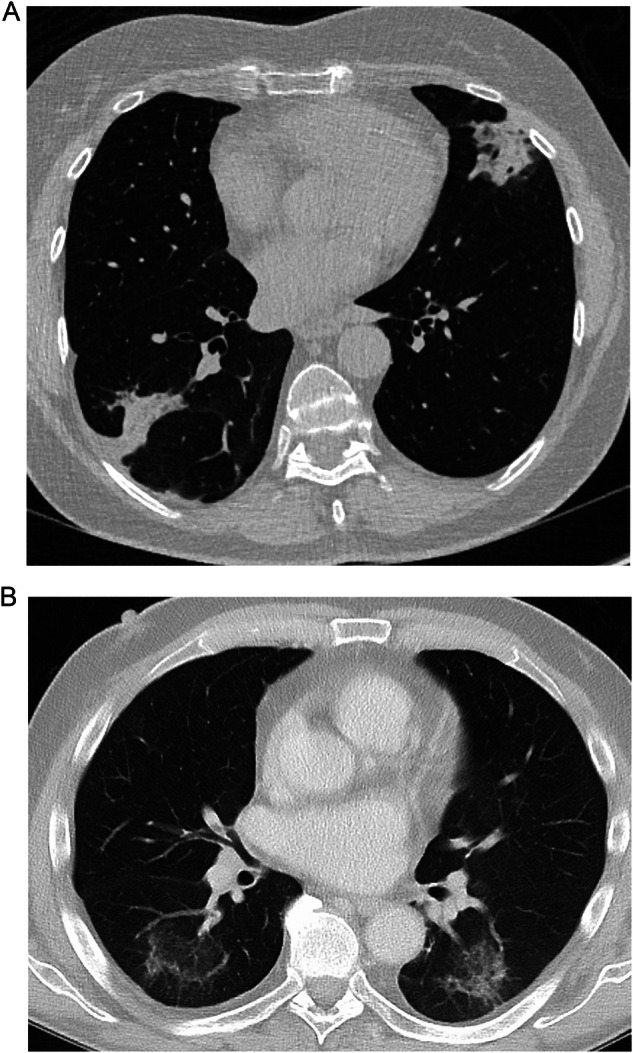
**A** CT scan of a patient with breast cancer and drug-induced ILD with OP pattern, characterized by sharply demarcated, bilateral, peripheral peribronchial consolidations (diagnosed by a multidisciplinary team [MDT] consensus); **B** CT scan of a patient with lung cancer and MDT-diagnosed drug-induced ILD with OP pattern and atoll sign in both under lobes. Images reproduced with permission from Dinkel J. 2023. University Hospital LMU Munich, Munich, Germany

The OP pattern is characterized by sharply demarcated, bilateral, peripheral consolidations that may be migratory, fluctuating in position over time [56, 58] (Fig. 1A). The reversed halo, also known as the ‘atoll sign’, where a dense outer rim of consolidation is situated around a focal ground-glass opacity (GGO), may also be observed with this pattern [58] (Fig. 1B).

Though radiological patterns for SACT-related ILD can be highly variable for the same drug [6], some ILD patterns may occur more frequently in patients being treated with a specific drug or drug class. A pooled analysis of 90 patients with advanced cancers and ILD associated with T-DXd across two Phase 1/2 single-arm, multicenter, global clinical trials found that 72.2% of cases (*n* = 65/90) had the OP pattern [59–61]. Similarly, a Japanese nationwide post-marketing surveillance program found that among 130 patients with HER2-positive metastatic breast cancer or gastric cancer and adjudicated T-DXd-related ILD, the majority of cases (63.1%) had the OP pattern [62]. The OP pattern was also the most common type observed in a retrospective multicenter study of patients with ILD related to treatment with ICIs, including cytotoxic T-lymphocyte-associated protein 4 (CTLA‑4) inhibitors, PD-1 inhibitors, and programmed cell death ligand 1 (PD-L1) inhibitors (23.4%, *n* = 15/64) [63].

##### HP pattern

**Fig. 2:**
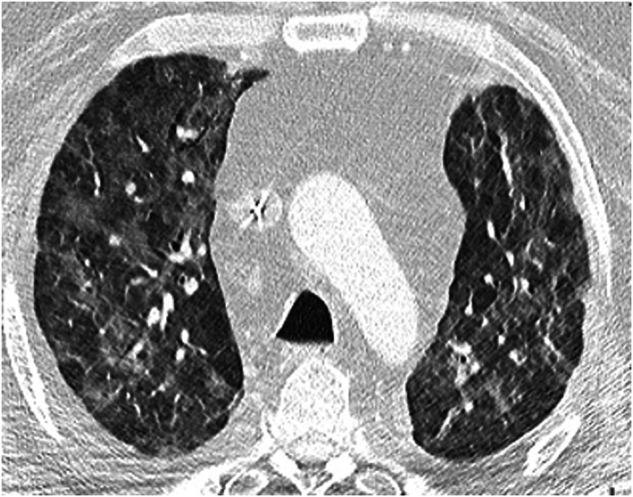
CT scan of a patient with systemic anticancer therapy-induced interstitial lung disease with the hypersensitivity pneumonitis pattern, diagnosed by multidisciplinary team consensus. The examinations were performed with contrast to exclude the presence of pulmonary embolism. Diagnosis of the three-density pattern is therefore difficult after injection. It is recommended that the examination be repeated without contrast agent if there is any uncertainty. This CT scan depicts mosaic density with extensive ground glass opacities and exclusion of several secondary lobules. Images reproduced with permission from Dinkel J. 2023. University Hospital LMU Munich, Munich, Germany

The HP pattern has a distinctive phenotype on HRCT [64]. Extensive GGOs can be observed, with areas of high attenuation and air trapping (low attenuation) forming a characteristic mosaic pattern, also known as ‘head cheese sign’ or the ‘three-density pattern’ [64, 65] (Fig. 2). Normal, hypodense, and hyperdense areas of lung parenchyma coexist on the HRCT scan [65]. Fibrotic changes can occur with the drug-induced HP pattern, but are rare in the context of SACT-induced toxicity.

A retrospective analysis of patients with advanced cancer enrolled into Phase 1 clinical trials in a single center found that HP was the most commonly occurring pattern among those with ILD related to treatment with phosphoinositide 3-kinase / AKT serine / threonine kinase / mTOR inhibitors, accounting for ~45.0% of all cases; this was closely followed by the NSIP pattern [66].

##### AIP/ARDS pattern

**Fig. 3:**
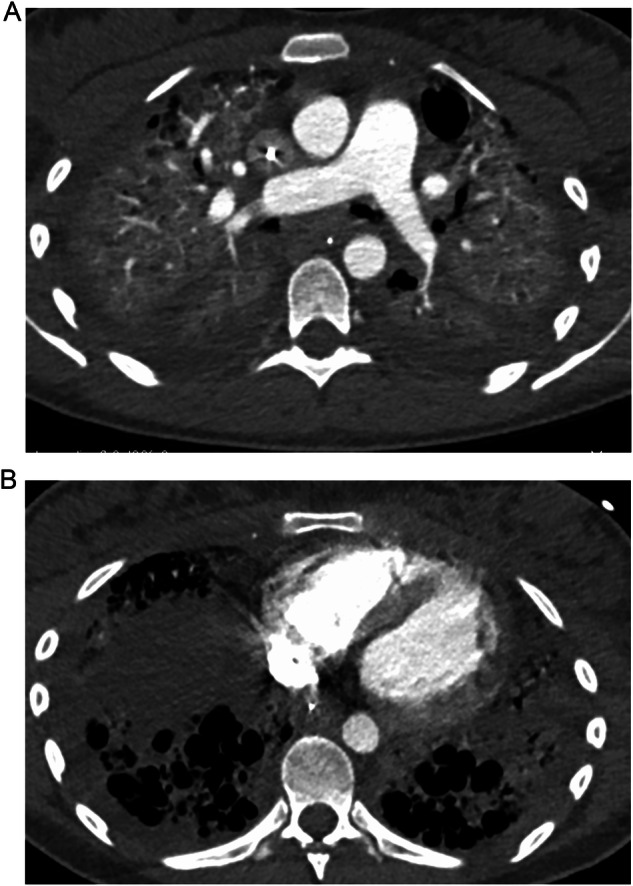
**A**, **B** CT scans of a patient with drug-induced interstitial lung disease showing the acute interstitial pneumonia / acute respiratory distress syndrome pattern, including bilateral consolidations and pleural effusion (diagnosed by multidisciplinary team consensus)

HRCT findings for the AIP/ARDS pattern include extensive areas of diffuse or patchy consolidation and GGOs, often with a crazy-paving appearance where areas of high attenuation resemble irregularly shaped paving stones [56, 67] (Fig. 3). Formal diagnosis requires pathological examination, if available, confirming the presence of diffuse alveolar damage (DAD) [56, 68]—a histological hallmark of the AIP/ARDS pattern [56, 68]. However, patients typically present with such a serious clinical picture that the potential benefits of bronchoscopy sampling may not outweigh the risk of complications [69, 70]. The AIP/ARDS pattern is considered an aggressive form with a high mortality rate [69, 71], progressing rapidly over the course of days or weeks [56, 69]. A single-center real-world analysis of patients with SACT-related ILD requiring hospitalization (*N* = 120) found that the mortality rate was higher among patients with radiological patterns indicating DAD than among those with non-DAD patterns of drug-related ILD (53.3% vs. 13.3%, respectively); the presence of radiological patterns indicating DAD was associated with an increased risk of mortality compared with cases without DAD patterns (hazard ratio 11.24 [95% CI 4.82, 26.20], *p* < 0.01) [71].

#### Patterns with a reticulation HR pattern

##### NSIP pattern

**Fig. 4:**
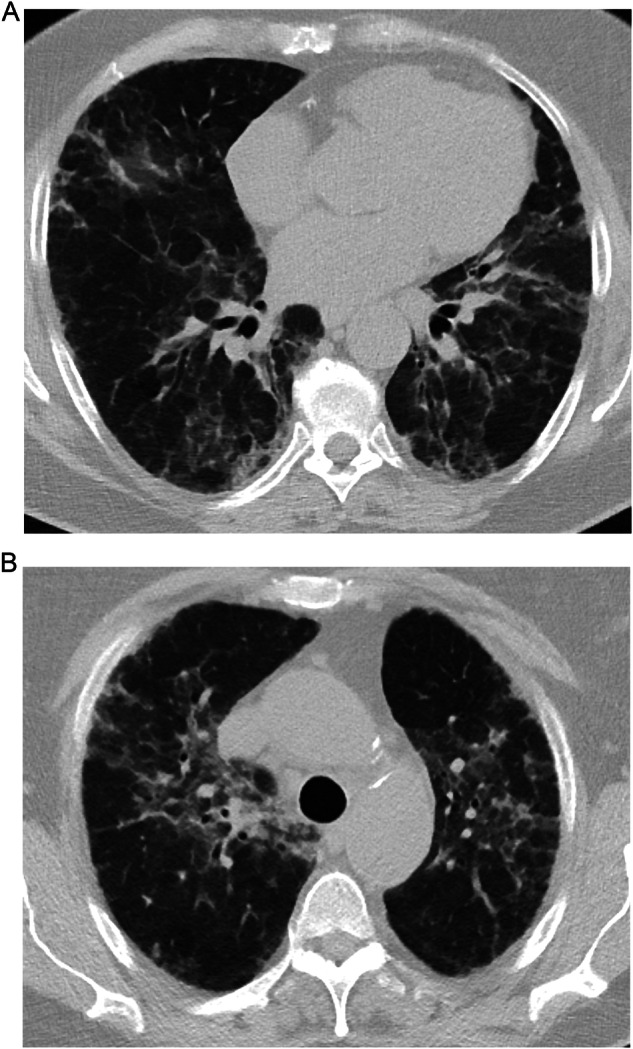
**A**, **B** CT scans of a patient with systemic anticancer therapy-induced interstitial lung disease showing the non-specific interstitial pneumonia pattern, diagnosed by multidisciplinary team consensus

The predominant findings for drug-induced ILD events with an NSIP pattern are usually symmetrical GGOs with peripheral, subpleural, and/or basal reticulation [56]. Secondly, the appearance of subtle traction bronchiectasis indicates a tendency towards fibrotic changes [72] (Fig. 4).

#### Rare forms of SACT-related ILD

##### Sarcoid-like reaction

**Fig. 5:**
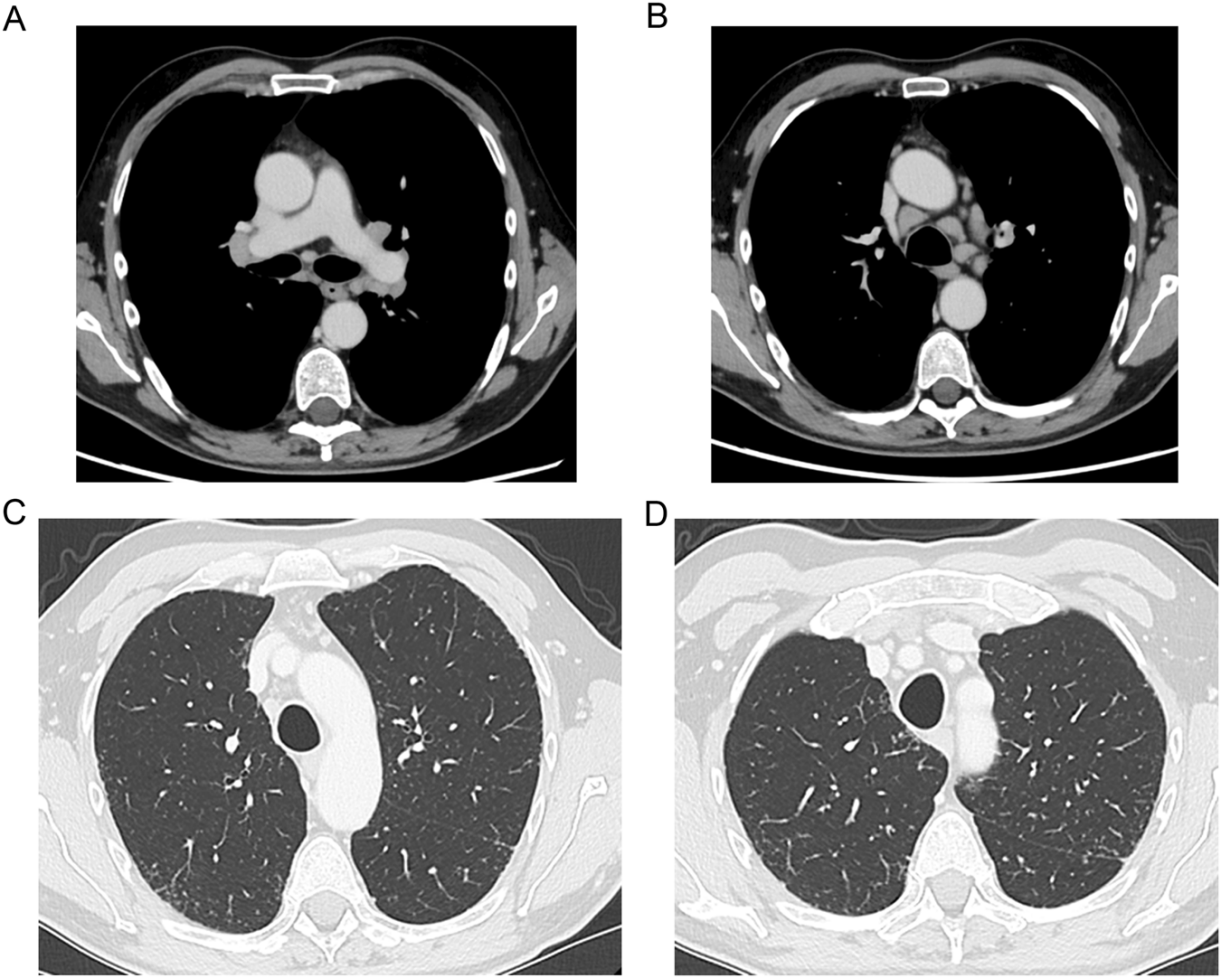
CT scans of a patient with a head and neck tumor and a sarcoid-like reaction induced by systemic anticancer therapy (diagnosed by multidisciplinary team consensus). **A**, **B** After two cycles of pembrolizumab treatment, the patient developed new mediastinal lymphadenopathy and (**C**, **D**) a new pulmonary peri-lymphatic micronodular pattern. Sarcoidosis was found in a mediastinal lymph node sample and the case was finally classified by the interstitial lung disease board as a sarcoid-like reaction

Certain SACTs—including the ICIs ipilimumab, nivolumab, and pembrolizumab—have been associated with a rare clinical manifestation known as a ‘sarcoid-like reaction’, which is characterized by non-necrotic granulomas (Fig. 5) [73–75]. These reactions are indistinguishable from sarcoidosis, and as the pathogenesis of sarcoidosis is not known, it is unclear whether SACTs cause sarcoidosis or simply simulate the syndrome [73].

##### Radiation recall pneumonitis

**Fig. 6:**
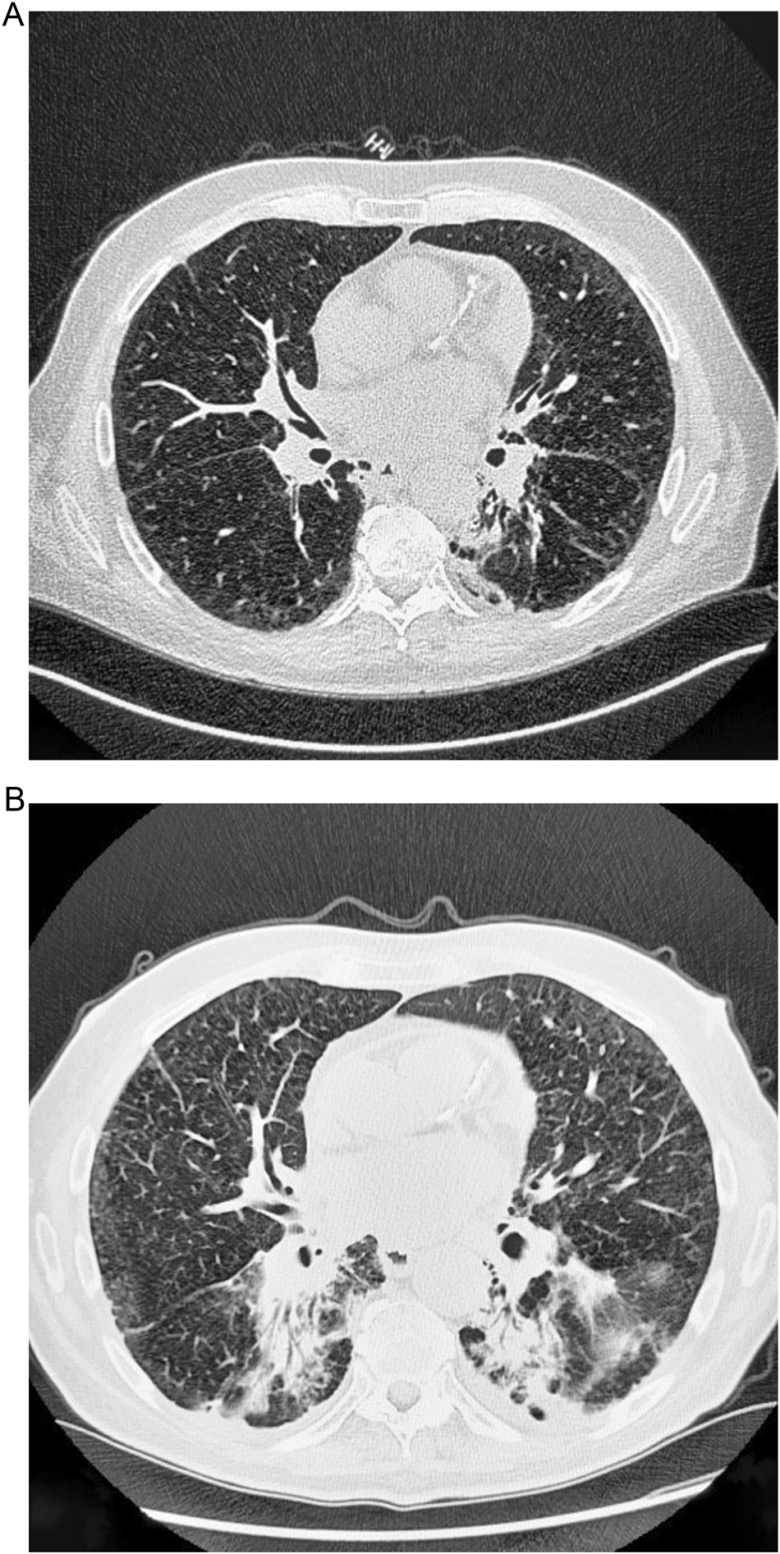
**A** CT scan of a patient with left-central lung cancer treated with definitive radiochemotherapy. Due to a recurrence 2 years later in the form of mediastinal lymphadenopathy, the patient was treated with monoimmunotherapy and after four cycles the patient developed recall pneumonitis; **B** the diagnosis of which was established by consensus within a multidisciplinary team. Images reproduced with permission from Dinkel J. 2023. University Hospital LMU Munich, Munich, Germany

Radiation recall pneumonitis (RRP) is a rare reaction occurring in previously irradiated pulmonary tissue following exposure to ‘triggering agents’ (Fig. 6) [76], where areas of pneumonitis, characterized by consolidation or GGOs, are limited to the prior field(s) of radiation [77]. RRP has been observed following radiation and treatment with the ADC T-DXd, the anti-PD-1 drugs nivolumab and sintilimab, epidermal growth factor receptor (EGFR) TKIs, and various classes of chemotherapeutic agents [62, 78–81]. A retrospective study reviewed the medical records of patients with advanced non-small lung cancer who had received EGFR-TKIs within 5 years of radiotherapy and found that the RRP pattern was present in 6/20 (30.0%) patients who developed SACT-related ILD [78]. A Japanese nationwide post-marketing surveillance study found that, among 130 cases adjudicated as T-DXd-related ILD/pneumonitis in patients with breast and gastric cancer, 3/130 (2.3%) cases had ‘other’ imaging patterns, including RRP pattern, non-cardiogenic pulmonary edema pattern and cases where a definitive diagnosis of ILD/pneumonitis could not be made because of very light shadows [62].

### Differential diagnosis of SACT-related ILD

ILD is a diagnosis of exclusion; as radiological findings may be similar between SACT-related ILD and events secondary to other etiologies, they must be analyzed in combination with investigations from across the MDT [7, 15, 82]. Additionally, as not all ILD events in patients who are receiving SACT are drug-related, the diagnostic process should include identifying the cause of any ILD events detected, ensuring that the patient receives the most appropriate treatment.

Differential diagnoses of SACT-related ILD include the progression of an underlying disease, edema, other pulmonary disorders of unknown origin, and seasonal and/or opportunistic infections in immunocompromised patients [7, 17]. Interstitial pulmonary edema may also present with rales or crackles, and thus should be investigated when SACT-related ILD is suspected based on chest auscultation findings [83]. Infectious etiologies of interstitial pneumonia may be viral (caused by pathogens such as adenovirus, cytomegalovirus, Epstein-Barr virus, influenza, respiratory syncytial virus or severe acute respiratory syndrome coronavirus 2), bacterial (caused by *Haemophilus*, *Streptococcus*, or *Pseudomonas* species), or fungal (caused by organisms such as *Pneumocystis jirovecii [PJ]*) [84–87]. Microbial and serological tests, such as polymerase chain reaction assays, may help to exclude possible infectious etiologies when SACT-related ILD is suspected [7]. Joint clinical practice guidelines from the Latin American Thoracic Association, ATS, ERS, and Japanese Respiratory Society suggest that performing bronchoalveolar lavage (BAL) cellular analysis may help distinguish the etiology of suspected ILD events [88]. Pulmonary lymphangitis carcinomatosa can have a similar appearance to ILD on HRCT, with diffuse GGOs [56, 89, 90]. BAL and transbronchial biopsies can confirm the absence of adenocarcinoma cells [89]. Guidelines developed by the British Thoracic Society in collaboration with the Thoracic Society of Australia and New Zealand and the Irish Thoracic Society recommend excluding pulmonary embolism by CT pulmonary angiography when suspected ILD presents with respiratory failure [91]. In cases where there is uncertainty in confirming a diagnosis of ILD, HRCT imagery, test results, and patient details should be presented to the ILD board [92, 93].

The possibility for co-existing conditions should be considered when diagnosing SACT-related ILD. Although each potential differential diagnosis and co-existing condition has distinct diagnostic characteristics that aid in determining their likelihood, with CT imaging particularly valuable in this regard, ascertaining the primary limiting condition can be complex, especially when there is microbiological evidence of infection. For instance, the presence of PJ in the airways may not necessarily indicate Pneumocystis pneumonia (PJP), but simply colonization [94]. Usually, patients with PJP will present with a degree of hypoxia and respiratory distress [95], whereas SACT-induced ILD cases are typically mild in severity (Table 2). Although the co-existence of drug-induced ILD and PJP is rare [96], distinguishing between the two based solely on clinical presentation, imaging, and laboratory results can be difficult, particularly in severe cases [97, 98]. Where clear differentiation is challenging or time and resources are limited, it may be necessary to treat both conditions simultaneously. For example, it is not uncommon to administer steroids for SACT-related ILD and broad-spectrum antibiotics for suspected co-existing infections [94, 97]. Following the initiation of therapy, more detailed investigations typically guide definitive diagnosis.

SACT-related ILD may develop within days to months after drug administration; however, late clinical manifestations do occur and late-occurring ILD events should not be excluded as a possible diagnosis [18, 99].

### Future directions for detection of ILD

The use of artificial intelligence (AI) to detect patterns of ILD is increasingly being investigated, with existing literature demonstrating the ability of AI models to make crude classifications of suspected ILD cases, such as determining from HRCT scans whether cases are fibrosing or non-fibrosing, and classifying the pattern of ILD as ‘definitely’, ‘possibly’, or ‘inconsistent with’ usual interstitial pneumonia [100]. Before AI algorithms can be integrated into the process of detecting ILD and determining patterns of disease, they must be validated by expert radiologists [100]. Additionally, existing models rely solely on the results from HRCT scans; the development of multivariate models that can consider patient characteristics and clinical findings from MDT assessments may be the next approach investigated in the AI space [100].

There also appears to be interest in the identification of biomarkers that may help clinicians detect ILD [101, 102]. A single-center cross-sectional study of patients with ILD (*n* = 322) found that eNose technology demonstrated promising accuracy in distinguishing these patients from healthy controls (*n* = 48) through breath analysis (area under the curve 1.00), and may be a useful tool to increase diagnostic confidence in combination with the results of other clinical assessments [102]. Additionally, the use of wearable biosensors for detection of pulmonary dysfunction in high-risk individuals has also been investigated [103], and this could be further assessed in patients at high-risk of developing SACT-ILD.

### Therapeutic management of SACT-related ILD

Specific management guidelines are available for some SACTs that have an associated risk of lung injury, including post-marketing recommendations for the management of ILD events occurring during treatment with abemaciclib, everolimus, irinotecan, lapatinib, pembrolizumab, T-DM1, and T-DXd [12, 13, 26–30]. Corticosteroid administration is recommended for Grade ≥ 2 ILD events occurring during pembrolizumab treatment, with an initial dose of 1–2 mg/kg/day prednisolone or equivalent followed by taper [12]. Pembrolizumab should be withheld for Grade 2 events, with the option to resume in patients with complete resolution or partial resolution (to Grade 1) after corticosteroid taper; for recurrent Grade 2 ILD events and those Grade ≥ 3 in severity, pembrolizumab should be permanently discontinued [12]. A strategy for detecting, monitoring and managing T-DXd-related ILD, termed the five ‘S’ rules, has been developed, and includes *screening* (with careful patient selection prior to treatment initiation and regular clinical assessments to exclude signs/symptoms of ILD throughout treatment), *scanning* (with HRCT at baseline and repeated every 6–12 weeks), *synergy* (educating patients and the care team to facilitate early reporting of signs and symptoms, as well as multidisciplinary management once ILD is suspected), *suspending* treatment, and *steroid* treatment [101, 104]. On the diagnosis of a Grade 1 ILD event during treatment with T-DXd, the PI recommends a treatment delay, and rechallenge with a maintained dose if the event has completely resolved within 28 days of onset, or with the dose reduced one level if the event has resolved in greater than 28 days from onset [13]. In the investigators’ brochure for clinical trials, it is recommended that treatment with T-DXd be permanently discontinued if the Grade 1 ILD event has not resolved within 126 days of onset [105]. PI recommends that corticosteroid treatment should be considered as soon as ILD is suspected (e.g., ≥ 0.5 mg/kg/day prednisolone or equivalent) [13]. In the event of Grade ≥ 2 ILD, T-DXd treatment should be permanently discontinued and corticosteroid treatment promptly initiated (e.g., ≥ 1 mg/kg/day prednisolone or equivalent); this should be continued for at least 14 days followed by a gradual taper for at least 4 weeks [13]. Hospitalization is generally required for severe cases (Grade ≥ 3) [9]. Permanent discontinuation is recommended upon diagnosis of an ILD event of any grade related to treatment with irinotecan [26], and Grade ≥ 3 events related to treatment with lapatinib [27].

## Conclusion

As the cancer treatment landscape evolves, it is likely that the incidence of potentially life-threatening SACT-related ILD events will increase. Collaborative working of MDTs is imperative for accurate and timely detection, diagnosis, and management of SACT-related ILD, with evaluations by the radiologist, primary physician, nurse practitioner, pulmonologist, thoracic surgeon, pathologist, and infectious disease specialist, where appropriate, necessary to rule out other causes [7, 15].

Radiologists are at the forefront of asymptomatic, Grade 1 ILD detection. Proactive communication between treating physicians and radiologists, noting when patients are receiving SACTs with a known risk for ILD on scan requisitions and highlighting when ILD is suspected, makes early detection more feasible. The correct scan methodology and urgent reporting to the patient’s treating physician and allied team are paramount. Given its high sensitivity and specificity, HRCT with < 2-mm slice thickness is the most appropriate CT technique for the investigation of suspected ILD. A stepwise approach to ILD detection, underpinned by an awareness of high-resolution ILD patterns, which can impact patient prognosis, is crucial for accurate diagnosis. Where there is uncertainty in confirming a diagnosis of ILD, the case should be presented to the ILD board. Patients with confirmed SACT-related ILD should be monitored until complete resolution of the AE.

Early detection of SACT-related ILD allows prompt implementation of drug-specific treatment management guidelines and recommendations. Detection and diagnosis at Grade 1, before symptom onset and prior to redosing, allows time for resolution of ILD and will likely reduce the risk of progression to higher-grade lung toxicity. Additionally, identification and resolution at Grade 1 allows patients to be rechallenged with some SACTs, as per specific post-marketing recommendations, widening patients’ eligibility for future cancer treatment options.

## Abbreviations

ADC: Antibody-drug conjugate
AIP/ARDS: Acute interstitial pneumonia / acute respiratory distress syndrome
ATS: American Thoracic Society
BAL: Bronchoalveolar lavage
CDK4/6i: Cyclin-dependent kinase 4/6 inhibitor(s)
CTCAE: Common Terminology Criteria for Adverse Events
DAD: Diffuse alveolar damage
EGFR: Epidermal growth factor receptor
ERS: European Respiratory Society
GGO: Ground-glass opacity
HER2: Human epidermal growth factor receptor 2
HP: Hypersensitivity pneumonitis
HRCT: High-resolution computed tomography
ICI: Immune checkpoint inhibitor
ILA: Interstitial lung abnormality
ILD: Interstitial lung disease
MDT: Multidisciplinary team
mTOR: Mammalian target of rapamycin
NSIP: Non-specific interstitial pneumonia
OP: Organizing pneumonia
PD-1: Programmed cell death protein 1
PD-L1: Programmed cell death ligand 1
PI: Prescribing information
PJ: *Pneumocystis jirovecii*
PJP: Pneumocystis pneumonia
RRP: Radiation recall pneumonitis
SACT: Systemic anticancer therapy
T-DM1: Trastuzumab emtansine
T-DXd: Trastuzumab deruxtecan
TKI: Tyrosine kinase inhibitor
